# Successful Deceased Donor Liver Transplantation with Median Sternotomy for Budd–Chiari Syndrome: A Case Report and Review of the Literature

**DOI:** 10.70352/scrj.cr.24-0165

**Published:** 2025-04-25

**Authors:** Takahiko Omameuda, Yukihiro Sanada, Yasunaru Sakuma, Yasuharu Onishi, Taiichi Wakiya, Noriki Okada, Yuta Hirata, Toshio Horiuchi, Kiichiro Takadera, Ryosuke Akimoto, Tomoya Uehara, Naohiro Sata

**Affiliations:** Division of Gastroenterological, General and Transplant Surgery, Department of Surgery, Jichi Medical University, Tochigi, Japan

**Keywords:** Budd–Chiari syndrome, deceased donor liver transplantation, large-for-size graft, median sternotomy, thrombus migration

## Abstract

**INTRODUCTION:**

When a thrombus extends to the suprahepatic inferior vena cava (IVC) in patients with Budd–Chiari syndrome (BCS) requiring liver transplantation (LT), there is a risk of thrombus migration during hepatectomy that can potentially lead to pulmonary embolism. Intraoperative pulmonary embolism can be life-threatening and may necessitate urgent thrombectomy. However, preventive strategies for pulmonary embolism during LT in BCS cases with IVC thrombosis have seldom been discussed in the literature. We report a case involving a 51-year-old woman with BCS complicated by thrombi extending into the suprahepatic IVC who underwent deceased donor LT (DDLT) for acute liver failure (ALF).

**CASE PRESENTATION:**

A previously healthy 51-year-old woman with ALF secondary to BCS was admitted to our hospital. 19 days back, BCS was diagnosed at another hospital, where computed tomography revealed thrombi in the hepatic veins and IVC. She subsequently developed grade II hepatic encephalopathy and severe liver dysfunction. Conservative treatment was ineffective, and 4 days before the current admission, she experienced grade III hepatic encephalopathy and showed hepatofugal portal flow on ultrasonography. DDLT was performed on day 13 after admission. Median sternotomy was performed to clamp the suprahepatic IVC near the right atrium, mitigating the risk of thrombus migration during hepatectomy and allowing for urgent thrombectomy in case of pulmonary embolism. Additionally, because a large-for-size graft was being used, the median sternotomy enhanced visibility and provided adequate space, facilitating suprahepatic IVC anastomosis. Postoperatively, the patient experienced no complications related to the sternotomy and was discharged 58 days after surgery.

**CONCLUSIONS:**

This case report highlights the potential utility of median sternotomy during LT for BCS, particularly for cases with concerns regarding thrombus migration from the suprahepatic IVC, the need for rapid thrombectomy in the event of pulmonary embolism, and anticipated challenges in suprahepatic IVC anastomosis due to large-for-size grafts.

## Abbreviations

ALF
: acute liver failure
BCS
: Budd–Chiari syndrome
DDLT
: deceased donor liver transplantation
GRWR
: graft versus recipient body weight ratio
IVC
: inferior vena cava
LT
: liver transplantation
SVC
: superior vena cava

## INTRODUCTION

Budd–Chiari syndrome (BCS) is a rare disorder characterized by obstruction of hepatic venous outflow.^1)^ Liver transplantation (LT) for BCS is performed only in select situations such as acute liver failure (ALF).^2)^ Inferior vena cava (IVC) reconstruction is required in LT. In cases where a thrombus extends to the suprahepatic IVC, there is a risk of thrombus migration during hepatectomy, which may lead to pulmonary embolism. Although intraoperative pulmonary embolism can be fatal and may require urgent thrombectomy,^3)^ preventive measures against pulmonary embolism during LT in cases of BCS with IVC thrombosis are rarely addressed in the literature.

With regard to LT for BCS, there are very few reports on the use of a median sternotomy approach to the suprahepatic IVC; intra-abdominal and transdiaphragmatic approaches seem to be more commonly employed. Here, we report a case involving a 51-year-old woman with BCS complicated by suprahepatic IVC thrombosis who successfully underwent deceased donor LT (DDLT) with median sternotomy. We also present a review of the relevant literature.

## CASE PRESENTATION

A previously healthy 51-year-old woman was transferred to our hospital with ALF due to BCS. 19 days before her current admission, BCS was diagnosed at another hospital following a computed tomography (CT) scan that revealed thrombi in the hepatic veins and IVC. She was admitted and started on heparin therapy. 16 days before her current admission, she developed grade II hepatic encephalopathy and exhibited severe liver dysfunction, leading to a diagnosis of ALF due to BCS. Conservative treatment was ineffective, and 4 days before her current admission, she experienced grade III hepatic encephalopathy and showed hepatofugal portal flow on ultrasound. Therefore, she was admitted to our hospital for consideration of emergency LT, with a diagnosis of ALF due to BCS. On the day of admission, blood test results showed the following: pH, 7.457 (range 7.35–7.45); serum alanine aminotransferase, 78 IU/L (range 7–23 IU/L); aspartate aminotransferase, 78 IU/L (range 13–30 IU/L); total bilirubin, 5.59 mg/dL (range 0.40–1.50 mg/dL); platelet count, 68 × 10^3^/μL (range 158–348 × 10^3^/μL); prothrombin time, 19.0 s (range, 10.3–12.7 s); international normalized ratio, 1.7 (range 0.85–1.15); creatinine, 0.46 mg/dL (range 0.46–0.79 mg/dL); and ammonia, 187 μmol/L (range 11–35 μmol/L). The Model for End Stage Liver Disease score was 19.

**Fig. 1:**
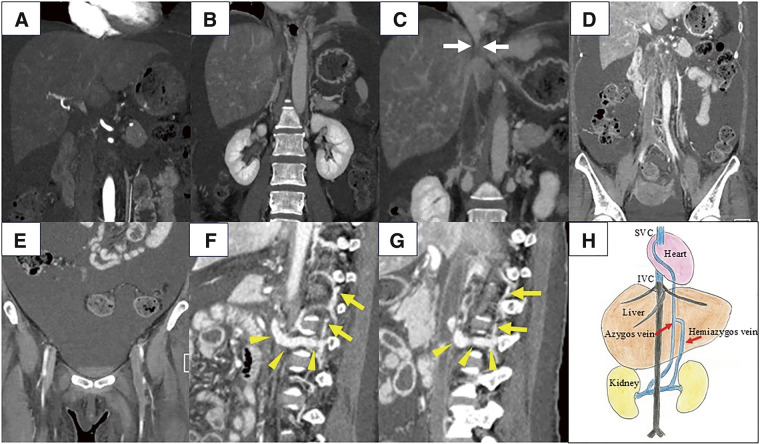
Preoperative images of a 51-year-old woman with acute liver failure secondary to Budd–Chiari syndrome. (**A**–**E**): coronal sections, (**F**, **G**): sagittal sections. (**A**) Computed tomography shows areas of diffuse early enhancement in the liver parenchyma and a large amount of ascites. (**B**) Thrombi are present in the major hepatic veins. (**C–E**) The thrombi in IVC are extending from the suprahepatic IVC (white arrows) to the veins in both lower extremities. (**D**, **E**) Blood flow distal to the renal veins can be seen entering SVC via well-developed azygos veins. (**F**) Blood flow from the left renal vein is directed to the hemiazygos vein (yellow arrows) via the communicating vein (yellow arrowheads). (**G**) Blood flow from the right renal vein is directed to the azygos vein (yellow arrows) via the communicating vein (yellow arrowheads). (**H**) The schema, prepared by the authors, shows the location of the thrombi, and the blood flow from both renal veins is sufficient to enter SVC via the azygos veins. IVC, inferior vena cava; SVC, superior vena cava

**Figure 1** shows the findings on contrast-enhanced CT performed on admission. There were areas of diffuse early enhancement in the liver parenchyma and a large amount of ascites (**Fig. 1A**). Thrombi were present in the hepatic veins and IVC (**Fig. 1B**, **1C**), with the thrombi in IVC extending from the suprahepatic IVC to the veins in both lower extremities (**Fig. 1D**, **1E**). IVC was not completely occluded, but the blood flow distal to the renal veins entered the superior vena cava (SVC) via well-developed azygos veins (**Fig. 1F**, **1G**). CT and Doppler ultrasound confirmed patency of the portal vein and hepatic artery.

Given the lack of effective interventions for ALF, LT is considered a necessary life-saving measure. We promptly initiated anticoagulant therapy and registered the patient with the Japan Organ Transplant Network on day 2 of admission, with the hope of performing DDLT.

After admission, thrombocytopenia further progressed, with the platelet count decreasing to 42 × 10^3^/μL on day 8. A thorough evaluation for BCS revealed no JAK2 gene mutations or evidence of myeloproliferative disorders. However, on day 12 after admission, heparin-induced thrombocytopenia (HIT) antibodies tested positive during the investigation of thrombocytopenia. Considering the clinical course from the previous hospital, a diagnosis of HIT was not established. The anticoagulant was switched from heparin to argatroban.

Preoperatively, we anticipated an anastomosis between the recipient suprahepatic IVC and the graft IVC. Clamping the suprahepatic IVC near the right atrium (RA) was necessary; however, attempting to do so from within the abdominal cavity posed a risk of thrombus migration and subsequent pulmonary embolism. To prepare for the possibility of such an event, which would require urgent thrombectomy, we decided to perform median sternotomy in advance; this would allow safe clamping of the suprahepatic IVC. A sheath was inserted into the right internal jugular vein, and the right groin was sterilized to facilitate sheath insertion if necessary, in preparation for the potential use of extracorporeal membrane oxygenation or cardiopulmonary bypass in the event of a pulmonary embolism. Intraoperatively, no specific examination for pulmonary thrombi was performed; instead, careful monitoring of vital signs was prioritized. However, if pulmonary thrombi were suspected, diagnostic tests such as transesophageal echocardiography or contrast-enhanced X-ray imaging would be performed promptly. In the event of a confirmed pulmonary embolism, cardiovascular surgeons would surgically remove the thrombus. We planned to promptly clamp the suprahepatic IVC by performing median sternotomy. Although CT showed that IVC was not completely occluded, blood flow from both renal veins was sufficiently entering SVC via the azygos veins (**Fig. 1H**). Therefore, we decided to transect the suprarenal IVC.

**Fig. 2:**
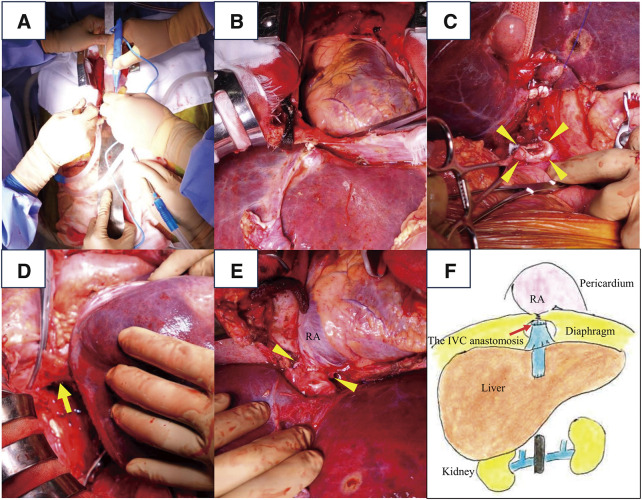
Intraoperative images and illustrated technical highlights for DDLT in a case of Budd–Chiari syndrome. (**A**) Midline and transverse laparotomies are performed, followed by median sternotomy. (**B**) The pericardium is incised, and the suprahepatic IVC is clamped near RA. (**C**) IVC is dissected caudally and divided at the suprarenal IVC (yellow arrowheads). (**D**) The suprahepatic IVC (yellow arrow) is clearly exposed. (**E**) End-to-end anastomosis (yellow arrowheads) is performed between the recipient’s suprahepatic IVC and the graft’s suprahepatic IVC. The schema shows the condition before abdominal and thoracic closure (excluding portal vein anastomosis, arterial anastomosis, and bile duct anastomosis). (**F**) The diaphragm is partially closed while the pericardium is kept open. DDLT, deceased donor liver transplantation; IVC, inferior vena cava; RA, right atrium

**Fig. 3:**
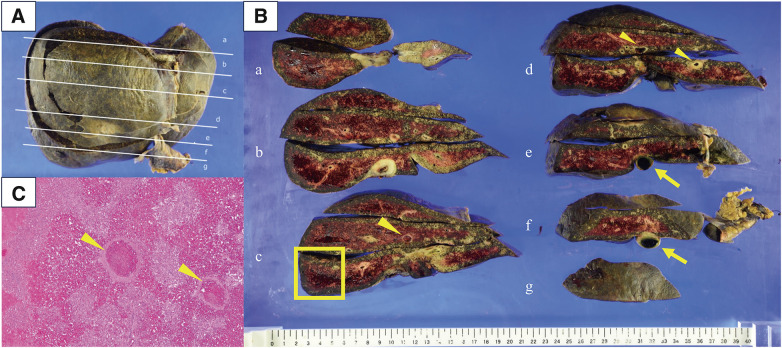
Pathological findings in the resected liver tissue. (**A**–**B**) The resected liver was divided into segments **a**–**g**, with thrombosis observed in the IVC (yellow arrows) and hepatic veins (yellow arrowheads). (**C**) indicates the area within the yellow square in (**B**). Hematoxylin and eosin staining (×4) revealed necrosis and hemorrhage, with dilation of the sinusoids. A thrombus (yellow arrowheads) was observed within the hepatic veins (**C**). IVC, inferior vena cava

DDLT was performed on day 13 after admission. The patient underwent midline and transverse laparotomies, followed by median sternotomy (**Fig. 2A**). The pericardium was then incised, and the suprahepatic IVC was clamped near RA (**Fig. 2B**). The diaphragm was split to expose the suprahepatic IVC. IVC was dissected caudally and divided at the suprarenal IVC, as planned preoperatively (**Fig. 2C**). Subsequently, the suprahepatic IVC (**Fig. 2D**) was divided and the native liver was removed. End-to-end anastomosis was performed between the recipient’s suprahepatic IVC and the graft’s suprahepatic IVC (**Fig. 2E**). Then, end-to-end anastomosis was performed between the recipient’s main portal vein and the graft’s main portal vein. End-to-end anastomosis was also performed between the recipient’s right hepatic artery and the graft’s gastroduodenal artery, and between the recipient’s left hepatic artery and the graft’s left gastric artery. In addition, end-to-end anastomosis was performed between the recipient’s common hepatic duct and the graft’s common bile duct. The diaphragm was partially closed, followed by closure of the abdominal and thoracic walls (**Fig. 2F**). The graft comprised a whole liver weighing 1791 g, with a graft versus recipient body weight ratio (GRWR) of 2.95%. The total surgical duration was 564 min, and the intraoperative blood loss was 9250 mL. In total, 26 units of red blood cells were transfused, and the cold ischemia time was 9 h and 37 min. The surgery was completed without active venovenous bypass. Macroscopic and histological examination of the explanted liver revealed thrombotic occlusion of the IVC or hepatic veins, along with congestive changes, which are consistent with the diagnosis of BCS (**Fig. 3**).

**Fig. 4:**
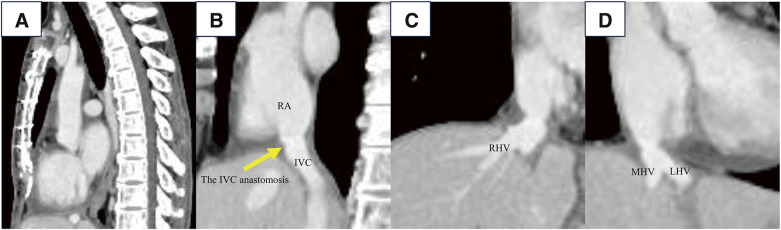
Computed tomography images at 3 months postoperatively. (**A**): sagittal section. (**B**–**D**): coronal sections. (**A**) The computed tomography scan performed 3 months postoperatively revealed no findings within the mediastinum, and (**B**–**D**) no thrombi were detected in the IVC or major hepatic veins. IVC, inferior vena cava; LHV, left hepatic vein; MHV, middle hepatic vein; RA, right atrium; RHV, right hepatic vein

Postoperatively, the patient had no complications associated with the median sternotomy, such as infection, bleeding, pain, or fracture and was discharged on postoperative day 58. The CT scan performed 3 months postoperatively revealed no findings within the mediastinum, and no thrombi were detected in the IVC or major hepatic veins (**Fig. 4**). More than 7 months after DDLT, she continued to take immunosuppressants (tacrolimus, mycophenolate mofetil, and methylprednisolone) and an anticoagulant (edoxaban). There was no recurrence of BCS.

## DISCUSSION

BCS is a rare clinical condition resulting from the obstruction of the hepatic venous outflow tract anywhere from the hepatic venules to the RA, including the small and large hepatic veins and IVC. When all three main hepatic veins draining the liver simultaneously clot, it can lead to diffuse and severe intrahepatic ischemia with massive necrosis and resultant ALF, as well as inadequate time for collaterals to develop. ALF is an uncommon presentation of BCS, while BCS is one of its rarest etiologies.^2)^ Early anticoagulation followed by transjugular intrahepatic portosystemic shunt and/or LT for ALF-BCS are likely the main modalities to be employed in managing patients with this disease. We performed a DDLT for ALF due to BCS with positive HIT antibodies. Although HIT prevalence in a cohort of patients with BCS is significantly high, no correlation was found between the rate of LT and HIT occurrence.^4)^ The primary cause of thrombocytopenia was considered to be ALF; however, the relationship between the positive HIT antibodies and BCS onset or progression remains unclear. Therefore, further accumulation of cases and research is necessary to better understand these conditions.

To the best of our knowledge, this is the first case of successful DDLT with median sternotomy in a patient with BCS. This case presents two clinically important findings.

First, we anticipated that the anastomosis between the recipient’s suprahepatic IVC and the graft’s IVC would be near RA; therefore, we opted for median sternotomy to allow safe clamping of the suprahepatic IVC and ensure urgent thrombectomy in the event of a pulmonary embolism. Two reported cases have involved the use of similar techniques, with anastomosis to either RA or the suprahepatic IVC (**Table 1**). Fukuda et al. performed median sternotomy to expose RA for anastomosis after construction of a mesoatrial shunt,^5)^ whereas Yoon et al. did so because the transdiaphragmatic approach alone did not provide sufficient space and view for clamping the right lateral side of RA.^6)^ Although there have been no reports of median sternotomy for LT in patients with BCS because of concerns regarding intraoperative thrombus migration, median sternotomy allows for rapid clamping of the suprahepatic IVC, which can prevent intraoperative pulmonary embolism and help prepare for urgent thrombectomy if needed. Ideally, clamping the inflow should have been performed first. However, in this case, we determined that clamping the IVC via median sternotomy could be achieved more quickly than identifying and clamping the portal vein in the abdominal cavity; therefore, we opted to clamp the IVC first. No thrombus was identified in the portal vein. Clamping the IVC did not result in intestinal congestion due to the presence of shunt vessels in the portal venous system.

**Table 1:** Summary of past reports on sternotomy in liver transplantation for Budd–Chiari Syndrome

No.	Author	Type of surgery	Sternotomy	The cause of sternotomy	IVC anastomosis	Complications associated with sternotomy	Prognosis
1	Fukuda et al.	LDLT	Median sternotomy	To expose the RA for anastomosis in a patient after the construction of a mesoatrial shunt	The recipient suprahepatic IVC and the graft left hepatic vein	Paroxysmal supraventricular tachycardia	Alive
2	Yoon et al.	LDLT	Lower median sternotomy	The transdiaphragmatic approach alone did not provide sufficient space and view for clamping the right lateral side of the RA	The right atrium and the interposing polyester graft	N/A	Alive
3	Our case	DDLT	Median sternotomy	To clamp the suprahepatic IVC, mitigating the risk of thrombus migration during hepatectomy and allowing for emergency thrombectomy in case of pulmonary embolism	The recipient suprahepatic IVC and the graft suprahepatic IVC	None	Alive

DDLT, deceased donor liver transplantation; IVC, inferior vena cava; LDLT, living donor liver transplantation; RA, right atrium

Second, the patient was a thin woman with a small chest and abdominal cavity, and a large-for-size graft was being used. These factors presented technical challenges, particularly for IVC anastomosis. Median sternotomy allowed for ample visibility and space, facilitating easier IVC anastomosis. During LT, a large-for-size graft can make suprahepatic caval anastomosis extremely difficult or even impossible.^7)^ However, in this case, the adequate visibility and space provided by the median sternotomy facilitated IVC anastomosis. Pu et al. diagnosed large-for-size graft when the GRWR exceeded 2.5%.^8)^ In this case, the GRWR was 2.95%, consistent with a diagnosis of large-for-size graft. Without a thoracotomy, IVC anastomosis would have been more challenging, potentially leading to prolonged warm ischemia time. For patients with a small chest and abdominal cavity and a large-for-size graft, median sternotomy may be advantageous in facilitating IVC anastomosis, thereby potentially reducing warm ischemia time.

However, the applicability of this technique requires careful evaluation. In cases where anastomosis to RA or the suprahepatic IVC is not required, or when the chest cavity is not small and the graft is not a large-for-size graft, it could result in excessive invasiveness. In a study by Yamada et al., in LDLT, the fibrous tissue surrounding the suprahepatic IVC was dissected to expose IVC in the posterior mediastinum, and a thin-bladed scissor was passed anterior to IVC to separate it cranially for 2–3 cm from the diaphragmatic crus to the intact suprahepatic IVC. Then, the diaphragm was adequately mobilized and dissected on the cranial side.^9)^ Several reports have described transdiaphragmatic approaches involving incision of the diaphragm and pericardium to clamp RA or the suprahepatic IVC.^10–14)^ In this case, we initially considered these approaches preoperatively but opted for median sternotomy under the assumption that urgent thrombectomy would be necessary in the event of thrombus migration during surgery. If RA or the suprahepatic IVC can be easily clamped using these techniques, median sternotomy is not necessary. Moreover, median sternotomy is considered very invasive if it is performed solely because of the presence of a large-for-size graft. Cases involving large-for-size grafts should be preferably managed using techniques such as graft segmentectomy, delayed fascial closure, and mesh closure.^8)^ Additionally, several complications such as infection, bleeding, pain, and fracture have been associated with median sternotomy in previous reports.^15,16)^ Although not observed in the present case, complications associated with median sternotomy should be carefully considered. This technique requires thorough preoperative assessment, as conducted in the present case, to determine its suitability for each case. If necessary, coordination with cardiovascular surgeons to discuss the possibility of sternotomy should be considered.

## CONCLUSIONS

We successfully performed DDLT using a median sternotomy approach for ALF in a patient with BCS complicated by thrombi in the suprahepatic IVC. In LT for BCS, combining median sternotomy increases surgical invasiveness, and its application should be carefully considered. However, in cases of LT for BCS with obstruction of the suprahepatic IVC, particularly cases where the chest and abdominal cavity are small and a large-for-size graft is available, this technique can prevent pulmonary embolism and enable rapid intervention in the event of an embolism. In addition, it facilitates easier IVC anastomosis, which can be complicated by a large-for-size graft, and helps reduce warm ischemia time, making it a valuable approach.

## ACKNOWLEDGMENTS

The authors thank Koji Kawahito and Hirohiko Akutsu from the Department of Cardiovascular Surgery, School of Medicine, Jichi Medical University; Shinya Otomo and Keiko Ogaki from the Department of Pharmacy, Jichi Medical University Hospital; and Sachiyo Yoshida and Natsumi Sekiya from the Transplantation and Regenerative Medicine Center, Jichi Medical University Hospital for their assistance with editing and formatting of the manuscript.

## DECLARATIONS

### Funding

No funding was received for this study.

### Authors’ contributions

TO contributed to the acquisition of clinical data and prepared the manuscript.

YS performed the surgery and perioperative management and contributed to drafting and revision of the manuscript.

YS supervised the decision-making process, implemented the treatment plan for the patient, and prepared the manuscript.

All the authors have read and approved the final version of this manuscript.

### Availability of data and material

All data supporting the conclusions of this study are included in this published article.

### Ethics approval and consent to participate

This case study was approved by the Ethics Committee of Jichi Medical University (Ethics Committee Approval Case No. 20-001). Informed consent for study inclusion or a substitute was obtained from the patients.

### Consent for publication

The patient and her family provided informed consent for publication of this report and accompanying images.

### Competing interests

The authors declare that there are no competing interests.
